# Long-Term Risk Trajectories of Diabetes Differ After Direct-Acting Antiviral and Interferon Therapy in Chronic Hepatitis C: A Real-World Cohort Study

**DOI:** 10.3390/biomedicines14061352

**Published:** 2026-06-15

**Authors:** Hsuan-Yu Hung, Wei-Liang Hung, Chung-Yu Chen

**Affiliations:** 1 School of Pharmacy, College of Pharmacy, Kaohsiung Medical University, Kaohsiung 80708, Taiwan; ameeyo36@gmail.com; 2 Department of Pharmacy, Ditmanson Medical Foundation Chia-Yi Christian Hospital, Chiayi 60002, Taiwan; 3 Department of Medicine, Division of Nephrology, Zuoying Armed Forces General Hospital, Kaohsiung 81342, Taiwan; lydership@gmail.com; 4 Master Program in Clinical Pharmacy, School of Pharmacy, Kaohsiung Medical University, Kaohsiung 80756, Taiwan

**Keywords:** hepatitis C, chronic, antiviral agents, direct-acting antivirals, interferon-alpha, diabetes mellitus, type 2, glycated hemoglobin A, sustained virologic response

## Abstract

**Background/Objectives:** Chronic hepatitis C (CHC) infection is an independent risk factor for developing type 2 diabetes mellitus (T2DM). However, it is unknown if antiviral treatment, especially with direct-acting antivirals (DAAs), changes long-term glycemic outcomes. **Methods:** We conducted a retrospective comparative cohort study of 2489 patients with chronic hepatitis C (CHC) in southern Taiwan between 2005 and 2022 who underwent treatment with either an interferon (IFN)-based or direct-acting antiviral agent (DAA) regimen. Given the distinct treatment eras of IFN and DAA therapies, potential temporal confounding was considered in the analytical design. Patients with existing diabetes or co-infections were excluded. The incidence of new-onset T2DM and longitudinal HbA1c levels were compared between treatment groups over a mean follow-up period of 2.56 years. **Results:** DAA-treated patients demonstrated a lower crude cumulative incidence of T2DM compared with IFN-treated patients (2.46% vs. 6.91%). However, adjusted analyses did not demonstrate a statistically significant difference between treatment groups. The cumulative risk appeared to plateau after the third year among DAA recipients. Post-therapy, HbA1c levels remained stable in both groups at between 5.5% and 6.5% over as long as five years. Splitting regression revealed that BMI ≥ 30 kg/m^2^, and not treatment type or achieved SVR, was an independent T2DM risk factor. The lowest rates of diabetes incidence were associated with pan-genotypic DAA regimens. **Conclusions:** DAA-treated patients showed lower crude T2DM incidence than IFN-treated patients; however, this difference was not consistently significant after adjustment for baseline factors. Viral eradication may be associated with favorable metabolic trends; however, the present findings do not establish a causal protective effect against incident T2DM. While increased BMI remained an independent predictor of post-treatment diabetes risk.

## 1. Introduction

Chronic hepatitis C virus (HCV) infection is now widely recognized as a systemic disease with metabolic consequences extending beyond hepatic injury. Among its extrahepatic manifestations, abnormalities in glucose metabolism—particularly insulin resistance (IR) and type 2 diabetes mellitus (T2DM)—have received increasing attention [1,2,3,4,5,6]. Epidemiological evidence consistently demonstrates a higher prevalence of T2DM in individuals with chronic HCV infection compared with the general population [1,2,4,7], suggesting a contributory role of viral infection in metabolic dysregulation. Proposed mechanisms include HCV-mediated impairment of insulin receptor substrate signaling, increased production of proinflammatory cytokines such as tumor necrosis factor-alpha (TNF-α), hepatic steatosis, and chronic immune activation, all of which may contribute to systemic insulin resistance and β-cell dysfunction [2,3,8,9].

Achievement of sustained virologic response (SVR) has become the central therapeutic goal of HCV management, not only to prevent hepatic complications but also to potentially ameliorate extrahepatic metabolic disturbances [1,10,11,12]. Both interferon (IFN)-based therapy and direct-acting antiviral (DAA) regimens can achieve SVR; however, they differ substantially in immunologic effects, treatment duration, and tolerability [13,14,15]. Although DAAs provide highly effective and well-tolerated viral eradication, whether their metabolic impact is comparable to or superior to IFN-based therapy remains uncertain [6,16,17]. Despite increasing evidence linking HCV eradication to improvements in glucose metabolism, direct comparisons between IFN- and DAA-based therapies regarding long-term T2DM risk remain limited. Because IFN and DAA therapies differ substantially in immunomodulatory effects and treatment era characteristics, whether the mode of antiviral therapy independently influences subsequent metabolic outcomes remains unclear.

While several studies have reported improvements in glycemic parameters following HCV clearance, direct comparisons between treatment modalities are limited [10,16,18,19]. In addition, the durability of glucose homeostasis after SVR remains controversial [16,17,20]. Given that T2DM significantly increases the risk of morbidity and healthcare burden in this population, understanding the long-term metabolic impact of antiviral therapy is of critical importance.

Therefore, we conducted a retrospective comparative cohort study to compare the long-term incidence of new-onset T2DM between patients treated with IFN-based and DAA-based regimens. The primary outcome was incident T2DM after antiviral therapy, while secondary outcomes included longitudinal HbA1c trends and virologic response patterns. By evaluating real-world outcomes across two distinct treatment eras, this study aimed to clarify whether the mode of viral eradication is associated with subsequent glucose metabolic abnormalities.

## 2. Materials and Methods

### 2.1. Ethics Statement

This study was conducted in accordance with the Declaration of Helsinki. The protocol was approved by the Institutional Review Board of Ditmanson Medical Foundation Chia-Yi Christian Hospital (DMF-CYCH) (IRB No. 2022062). Written informed consent was waived due to the retrospective nature of the study and the use of de-identified data.

### 2.2. Data Source

This retrospective comparative cohort study utilized data from the Ditmanson Research Database (DRD) in southern Taiwan. Patients aged ≥18 years with documented HCV infection who received IFN- or DAA-based therapy between 8 April 2005, and 30 June 2022 were identified through computerized administrative records.

### 2.3. Study Population

Participants aged ≥18 years with chronic HCV infection were categorized into an IFN-based cohort (8 April 2005–31 December 2020) and a DAA-based cohort (15 April 2016–31 December 2020). Follow-up continued until 30 June 2022 to ensure at least 15 months of post-treatment observation. All patients completed antiviral therapy and underwent SVR testing. Baseline characteristics were assessed using data obtained during the year preceding antiviral initiation (Figure S1).

Patients with prior antiviral exposure, antidiabetic medication use, or pre-existing diabetes during the washout period were excluded. Additional exclusions included acute HCV infection, alcoholic hepatitis, HBV or HIV co-infection, and liver or renal transplantation. Diagnostic codes are provided in Table S1 [21,22,23,24]. No formal sample size calculation was performed, as this retrospective cohort included all eligible patients during the predefined study period.

### 2.4. Outcome Measurement

The primary outcome was incident T2DM. Follow-up began at the end of antiviral therapy and continued until T2DM diagnosis [19] or study termination. T2DM was defined by either: (1) ≥2 outpatient or inpatient claims with International Classification of Diseases, 9th Revision, Clinical Modification (ICD-9-CM) 250.- or ICD-10-CM E11.-, or (2) one diagnostic claim accompanied by prescription of an antihyperglycemic agent.

Secondary outcomes included virologic response categorized as SVR12/24 (HCV RNA < 15 IU/mL), virologic failure, or relapse [25]. Long-term glycemic control was evaluated using serial glycated hemoglobin (HbA1c) measurements collected at 90-day intervals.

### 2.5. Subgroup Analysis

A subgroup analysis was also performed to assess the association between virological outcomes and T2DM incidence across different liver conditions. Patients were stratified into three subgroups based on liver status: (1) normal liver function, (2) liver fibrosis, and (3) liver cirrhosis. Within each subgroup, T2DM incidence was further analyzed according to virologic response category to examine differential glycemic risk patterns related to treatment response in distinct hepatic conditions.

### 2.6. Sensitivity Analysis

Because IFN- and DAA-based therapies were administered during different clinical eras, sensitivity analyses using propensity score (PS) matching were conducted to minimize potential confounding related to temporal treatment differences. The PS Matching model included age, Body Mass Index [BMI], sex, digestive system neoplasms, liver tumor, PD, hyperlipidemia, CKD, hepatic fibrosis, HTN, and cirrhosis. Matching was conducted 1:1, and covariate balance was confirmed with SMD < 0.10. The primary outcome (T2DM cumulative incidence) was reanalyzed in the matched cohort to assess robustness against confounding by indication and cohort overlap.

### 2.7. Statistical Analyses

All analyses were conducted using SAS version 9.4 (SAS Institute Inc., Cary, NC, USA), with two-sided *p*-values < 0.05 considered statistically significant. To mitigate treatment selection bias, propensity scores were estimated via multivariable logistic regression incorporating baseline age, sex, BMI, and comorbidities. Inverse probability of treatment weighting (IPTW) for the average treatment effect [26] was applied, and covariate balance was assessed within the region of common support. IPTW and covariate balancing procedures were predefined analytical methods and therefore are described in this section rather than the Results. Covariate balance after weighting was evaluated using standardized mean differences (SMDs), with values <0.10 considered indicative of acceptable balance.

Time-to-event analysis for T2DM incidence was conducted using Cox proportional hazards models adjusted for HCV genotype 1b, with results reported as adjusted hazard ratios (HRs) and 95% confidence intervals (CIs). Poisson regression was used to estimate incidence rate ratios (IRRs), and cumulative incidence (%) was calculated over the follow-up period.

Categorical variables were compared using chi-square or Fisher’s exact tests; continuous variables were summarized as mean ± SD and analyzed by repeated measures ANOVA. Missing HbA1c values were imputed using the median of the nearest two values. Temporal trends in T2DM incidence were evaluated using the Cochran–Armitage trend test, and a two-level multivariable model assessed changes in glycemic status pre- and post-treatment.

## 3. Results

### 3.1. Patient Characteristics at Baseline

**Figure 1:**
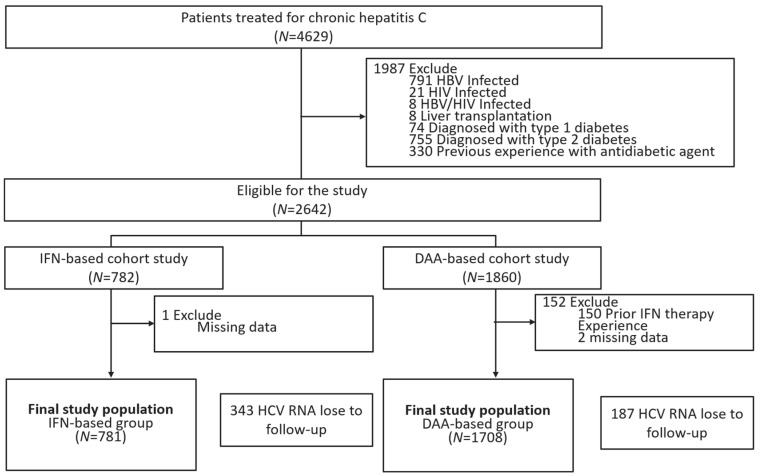
Patient enrollment and cohort allocation flowchart.

Of 4629 treated patients, 1987 were excluded according to predefined criteria, leaving 2642 eligible participants. After exclusion of patients with incomplete follow-up or missing covariate data, 2489 patients were included in the final analysis, comprising 781 IFN-treated and 1708 DAA-treated individuals. Both intention-to-treat and per-protocol analyses were performed (Figure 1).

### 3.2. Baseline Characteristics

The study cohort comprised 2489 patients with CHC, including 1708 individuals in the DAA-based treatment group and 781 in the IFN-based treatment group. The mean age was significantly higher in the DAA group compared with the IFN group (60.2 ± 13.0 vs. 57.3 ± 12.7 years, *p* < 0.001). Mean BMI was slightly but significantly lower in the DAA group (24.25 ± 3.94 kg/m^2^) than in the IFN group (24.96 ± 4.01 kg/m^2^, *p* = 0.02). Females were more prevalent in the DAA group (59.07%) relative to the IFN group (49.68%, *p* < 0.001). The demographic and clinical differences presented in Table 1 reflect pre-weighting baseline characteristics. Post-IPTW covariate balance is presented separately in Table S2.

The distribution of HCV GTs differed markedly between groups. GT 1b was more prevalent in the DAA cohort (33.68%) compared to the IFN group (4.74%, *p* < 0.001).

DAA-treated patients typically received ≤8 or 12 weeks of therapy, while IFN regimens were longer (*p* < 0.001). RBV use was largely restricted to the IFN group.

Comorbidities including hyperlipidemia, constipation, sleep disorders, anxiety, and peptic ulcer with hemorrhage were more prevalent in the IFN group (all *p* < 0.001), while CKD was more common in the DAA group (7.4% vs. 2.9%, *p* < 0.001). Other liver-related complications were similar.

Differences in medication usage during the 12 months preceding antiviral therapy were also observed. IFN patients more frequently used silymarin, acetaminophen, and proheparum (all *p* < 0.001); notably, acetaminophen use was 71.7% vs. 24.5%.

### 3.3. Incidence of Type 2 Diabetes

During the observation period, a total of 96 new-onset T2DM cases were identified, with 54 occurring in the IFN group and 42 in the DAA group. The overall mean time to diagnosis was 2.56 ± 1.86 years. The cumulative incidence in the total population was 3.86%. Crude incidence was higher in the IFN-treated group (6.91%) than in the DAA-treated group (2.46%); however, adjusted analyses did not demonstrate statistically significant differences between treatment groups (adjusted HR and IRR shown in Tables S3 and S4).

Stratified by liver function, among patients with cirrhosis, 1 T2DM event was identified (in the IFN group; none in the DAA group), corresponding to cumulative incidences of 7.14% and 0%, respectively, with an overall rate of 2.04%. In those with fibrosis, 23 cases were documented (11 IFN, 12 DAA), with cumulative rates of 11.22% and 5.77%. Among individuals with normal liver function, 72 cases emerged (42 IFN, 30 DAA), translating to cumulative incidences of 6.24% and 2.04% (Table 2).

### 3.4. Virological Response

Overall SVR rate was 68.94%. DAA therapy achieved substantially higher SVR (87.53%) compared with IFN (28.3%), with lower rates of virologic failure, relapse, and loss to follow-up (Figure S2).

### 3.5. Subgroup Analysis Results

#### 3.5.1. Type 2 Diabetes Risk by Liver Function and Virologic Outcome

In the full cohort (*N* = 2489), the overall incidence of T2DM was 3.86%, lower in the DAA group (2.46%) than in the IFN group (6.91%), though not statistically significant (IRR 0.97; 95% CI, 0.63–1.48; *p* = 0.97). Among those achieving SVR, incidence was 2.39% (1.87% DAA vs. 5.88% IFN; IRR 1.13; 95% CI, 0.57–2.37; *p* = 0.86). T2DM was rare in cases of virologic failure or relapse, with no significant differences observed (Table S3).

In patients with normal liver function (*N* = 2074), incidence was 3.36%, again lower in the DAA group (2.04%) than IFN (6.24%) without statistical significance (IRR 0.96; 95% CI, 0.60–1.52; *p* = 0.84). Among SVR achievers, incidence was 2.3% (1.81% DAA vs. 5.56% IFN; IRR 1.20; 95% CI, 0.58–2.44; *p* = 0.62).

In patients with fibrosis (*N* = 306), the overall T2DM incidence was 7.52%, with a higher rate in the IFN-based group (11.22%) than in the DAA-based group (5.77%), but this difference did not reach statistical significance (IRR 2.22, 95% CI, 0.73–7.10; *p* = 0.19). Among those achieving SVR, the incidence of T2DM was 3.03% (2.76% for DAA-based and 5.00% for IFN-based), with no significant difference between groups (IRR 2.71, 95% CI, 0.23–143.39; *p* = 0.70).

Among cirrhotic patients (*N* = 49), T2DM was rare (2.04% overall) and occurred only in the IFN-based group (7.14%), with no cases reported in the DAA-based group.

Across all subgroup analyses, DAA-based therapy demonstrated numerically lower crude T2DM incidence than IFN-based therapy. However, most subgroup comparisons did not achieve statistical significance, and several estimates were associated with wide confidence intervals, likely reflecting limited event numbers and reduced statistical precision. Therefore, these subgroup findings should be interpreted cautiously.

Table S4 summarizes the hazard ratios for incident T2DM stratified by liver function and virologic response. In the total cohort (*N* = 2489), patients treated with DAAs exhibited no statistically significant difference in T2DM risk compared to those receiving IFN-based therapy (adjusted HR: 0.84; 95% CI: 0.51–1.38; *p* = 0.49). Similarly, SVR status did not significantly impact the risk of T2DM development (adjusted HR: 1.29; 95% CI: 0.56–2.98; *p* = 0.55). The association between virologic failure and T2DM was inconclusive due to broad confidence intervals and a lack of statistical significance (adjusted HR: 1.02; 95% CI: 0.08–13.76; *p* = 0.99).

Among participants with normal hepatic function (*N* = 2074), DAA therapy was again not associated with a significant change in T2DM incidence compared to IFN (adjusted HR: 0.95; 95% CI: 0.54–1.69; *p* = 0.87), and SVR remained a non-significant factor (adjusted HR: 1.42; 95% CI: 0.57–3.52; *p* = 0.45). Virologic failure could not be evaluated in the multivariable model due to sparse event counts.

In patients with hepatic fibrosis (*N* = 306), although unadjusted analysis suggested a non-significant trend, the adjusted HR for DAA treatment versus IFN was 0.48 (95% CI: 0.17–1.41; *p* = 0.18), indicating no statistically meaningful association. SVR and virologic outcomes could not be assessed in this subgroup due to insufficient data.

No analyses were conducted for the cirrhosis subgroup (*N* = 49) because of the limited number of events, precluding any reliable estimation.

Figure S3 presents Kaplan–Meier survival curves comparing the incidence of T2DM between treatment groups. In the left panel, patients treated with DAA exhibited a numerically lower probability of developing T2DM over time compared to those receiving IFN-based therapy. The divergence in survival curves became more apparent beyond two years of follow-up.

In the right panel, among patients who achieved SVR, those treated with DAA still demonstrated a lower cumulative risk of T2DM than the IFN-SVR group. These Kaplan–Meier findings show divergent incidence trajectories; however, given that adjusted analyses did not demonstrate statistically significant differences, these observations should be regarded as descriptive trends rather than evidence of a superior protective metabolic effect of DAA therapy.

#### 3.5.2. Direct-Acting Antiviral Regimen

Cumulative incidence analyses (Table S5) showed a time-dependent increase in newly diagnosed T2DM over 5 years, with divergent trajectories by treatment group. In the overall cohort, T2DM incidence rose from 1.00% at year 1 to 3.62% at year 5 (*p* for trend < 0.001). In the IFN group, incidence increased steadily from 1.02% (year 1) to 6.15% (year 5) (*p* for trend < 0.001). In contrast, the DAA group rose from 1.00% (year 1) to 2.46% (year 3) and then remained essentially unchanged through year 5 (*p* for trend = 0.001). Between-group comparisons (chi-square) showed no difference at year 1 (*p* = 1.00), whereas separation became evident from year 3 (*p* = 0.0043) and was more pronounced by year 5 (*p* < 0.001), suggesting divergent incidence trajectories between treatment groups, although adjusted analyses did not demonstrate statistically significant differences. The apparent plateau in the DAA cohort may be partly attributable to a shorter effective follow-up window.

In regimen-specific analyses (Table 3), cumulative T2DM risk varied substantially across DAA combinations. Daclatasvir/asunaprevir had the highest incidence, reaching 16.67% by year 3 (*p* for trend < 0.001). Dasabuvir/ombitasvir/paritaprevir/ritonavir increased to 4.27% by year 3 (*p* < 0.001), while ledipasvir/sofosbuvir and elbasvir/grazoprevir reached 3.86% and 3.29%, respectively (both *p* < 0.001). In contrast, pan-genotypic regimens showed low event rates: glecaprevir/pibrentasvir was 1.00% by year 3 (*p* = 0.08) and sofosbuvir/velpatasvir remained 0.31% throughout follow-up (*p* = 0.17). Overall, between-regimen differences at year 3 were significant (*p* = 0.004), indicating heterogeneity in post-treatment T2DM risk across DAA regimens.

#### 3.5.3. Age and Body Mass Index Group

In age-based analysis, the reference group was patients aged 40 to <55 years. Compared to this group, the risk of T2DM did not significantly differ across other age strata. For example, in patients aged 55 to <65 years, the univariable HR was 1.70 (95% CI: 0.97–2.99; *p* = 0.52), with a multivariable adjusted HR of 1.13 (95% CI: 0.48–2.68; *p* = 0.78). In those ≥75 years, the univariable HR was 1.97 (95% CI: 0.96–4.07; *p* = 0.06), and the adjusted HR was 1.44 (95% CI: 0.53–3.92; *p* = 0.48). No age group showed a statistically significant association with T2DM after adjusting for potential confounders (Table S6).

In contrast, BMI showed a significant association with diabetes risk. Using individuals with BMI 18.5 to 24 kg/m^2^ as the reference, those with BMI ≥ 30 kg/m^2^ had a significantly higher T2DM risk, with a univariable HR of 2.47 (95% CI: 1.08–5.61; *p* = 0.03) and an adjusted HR of 2.63 (95% CI: 1.15–6.01; *p* = 0.02). Patients in the overweight range (BMI 25 to <30 kg/m^2^) also had elevated risk, though not statistically significant (adjusted HR: 1.40, 95% CI: 0.73–2.69; *p* = 0.31).

These findings indicate that elevated BMI, particularly ≥30 kg/m^2^, is an independent predictor of incident T2DM, whereas age alone does not appear to significantly influence risk after adjustment.

### 3.6. Sensitivity Analysis Results

In the propensity score-matched cohort (*N* = 864), cumulative incidence of T2DM remained comparable between treatment groups. The overall incidence was 5.79%, with 6.41% observed in the IFN-based group and 5.35% in the DAA-based group. The HR for T2DM in DAA recipients compared to IFN recipients was 0.81 (95% CI: 0.43–1.53), suggesting no statistically significant difference in risk. These findings indicate that the primary outcome remained robust after accounting for potential confounding through PS matching and overlapping observation periods (Table S7).

### 3.7. Long-Term Glycemic Control Changes

**Figure 2:**
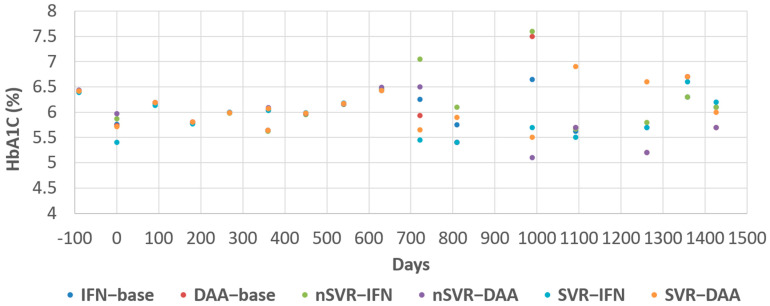
Trends in hemoglobin A1c after antiviral therapy according to treatment regimen and virologic response.

As shown in Figure 2, where Day 0 represents the final day of antiviral therapy, HbA1c levels across treatment and response groups appeared largely stable during both the treatment-free period and long-term follow-up. In the first 600 days post-treatment, most HbA1c values ranged between 5.5% and 6.5%, without significant upward or downward trends. This pattern was similar across IFN- and DAA-based therapies, regardless of virologic response.

Beyond 3 years, a few elevated HbA1c values (>7.0%) emerged, particularly in the nSVR-IFN group, suggesting possible delayed glycemic worsening in select patients. However, the magnitude of HbA1c change remained small across the cohort, and no sustained glycemic deterioration was observed in the SVR groups.

The overall minimal differences before and after treatment suggest that antiviral therapy alone may not substantially influence glycemic control for long-term benefit. It is also possible that residual or unmeasured confounding factors—such as diet, physical activity, or concurrent medications—contributed to the glycemic variability observed, particularly in non-SVR patients.

The predicted HbA1c trajectories derived from a two-level multivariable regression model comparing glycemic status pre- and post-antiviral therapy. Across all groups, a modest but consistent reduction in predicted HbA1c levels was observed following treatment initiation. This downward trend was present in both IFN- and DAA-based cohorts, regardless of SVR status. Although the overall decline in HbA1c was minimal (approximately 0.07% to 0.09%), the pattern suggests a potential glycemic benefit following antiviral therapy, albeit with limited clinical significance (Figure S4).

## 4. Discussion

In this retrospective two-cohort study of patients with CHC, DAA-treated patients exhibited a lower cumulative incidence of new-onset T2DM than IFN-treated patients (2.46% vs. 6.91%). Although Kaplan–Meier curves and cumulative incidence analyses suggested divergent long-term risk trajectories between treatment groups, adjusted Cox proportional hazards and IRR analyses did not demonstrate statistically significant differences. Therefore, these findings should be interpreted as observational associations and temporal trends rather than evidence of a causal protective metabolic effect attributable to DAA therapy. Furthermore, multivariable analyses adjusted for GT1b showed that neither antiviral treatment class nor achievement of SVR independently predicted incident T2DM, whereas elevated BMI (≥30 kg/m^2^) remained the strongest modifiable risk factor for diabetes development.

Longitudinal HbA1c trajectories were largely flat in both groups, with only small improvements, indicating that antiviral therapy exerts a limited glucose-lowering effect but appears metabolically safe with respect to long-term glycemic control. Within the DAA class, pan-genotypic regimens such as sofosbuvir/velpatasvir and glecaprevir/pibrentasvir were associated with the lowest T2DM incidence, whereas older non–pan-genotypic combinations showed higher risk. The concordance of results across intention-to-treat, per-protocol, and PS-matched analyses supports the robustness of these findings. Overall, our data suggest numerically lower T2DM rates with DAA—particularly modern pan-genotypic regimens—but these differences were not statistically significant after adjustment, and elevated BMI remained the strongest independent predictor of post-treatment diabetes risk, reinforcing the central role of metabolic risk factor management alongside antiviral therapy.

This retrospective study demonstrated a substantially lower cumulative incidence of new-onset T2DM in the DAA group than in the IFN group (2.46% vs. 6.91%), though adjusted analyses did not demonstrate a statistically significant difference between groups. These findings are broadly consistent with prior epidemiological data suggesting potential metabolic associations of HCV eradication [1,3,18,19,27,28]. Whereas untreated CHC and IFN-treated cohorts have shown persistently high annual T2DM incidence rates (approximately 20.6 and 19.8 per 1000 person-years, respectively) [29], the estimated annual incidence rate in DAA recipients in our cohort was markedly lower, at about 9.89 per 1000 PY, corresponding to an approximate 50% relative reduction in T2DM risk following viral eradication [10,29,30]. Furthermore, the cumulative incidence of T2DM in the DAA group appeared to stabilize and plateau after three years, whereas the crude rate in the IFN group continued to rise over time (*p* for trend <0.001 for both groups). This pattern suggests that long-term T2DM risk trajectories may differ between treatment eras, though the underlying mechanism and the extent to which this reflects a true treatment effect versus residual confounding remain uncertain [14,15,27,28,31]. Subgroup analyses further showed that pan-genotypic combinations such as sofosbuvir/velpatasvir (0.31% cumulative incidence) and glecaprevir/pibrentasvir (1.00%) were associated with the lowest T2DM risk, while non-pan-genotypic DAA regimens were linked to a significantly higher risk compared with pan-genotypic regimens; however, given the absence of statistical significance in adjusted models, these findings should be interpreted as hypothesis-generating rather than as evidence of a definitive metabolic protective effect [27,28,32,33,34,35,36].

Our results indicate that antiviral therapy for CHC, whether with IFN-based or DAA-based regimens, was not associated with significant long-term changes in blood glucose control. No significant differences were observed in the incidence of T2DM or in mean HbA1c levels before and after treatment. This finding is in line with a study from Brazil, which reported no significant change in HOMA-IR scores between baseline and 12 months after completion of HCV treatment [37].

In our cohort, neither the choice of IFN nor DAA regimen nor the achievement of SVR was associated with a significant reduction in T2DM risk after adjusting for age, sex, and BMI. This result is consistent with some prior studies, but stands in contrast to a larger body of literature demonstrating that successful antiviral therapy—especially SVR after IFN-based regimens—leads to significant improvements in glycemic control and a reduced risk of T2DM [18,30,38,39]. A recent meta-analysis further confirmed that HCV eradication can reduce both the risk of T2DM onset and diabetes-related complications [40].

Subgroup analyses in our study showed that the cumulative incidence of T2DM after SVR was lowest among patients treated with pan-genotypic DAA combinations such as sofosbuvir/velpatasvir and glecaprevir/pibrentasvir, consistent with findings suggesting a greater metabolic protective effect of these regimens [1,41]. However, the differences between regimens did not always reach statistical significance, likely due to sample size limitations. Notably, hepatic fibrosis and cirrhosis did not independently predict T2DM risk in our cohort, which is in contrast to reports that identify fibrosis as a critical driver of diabetes via chronic inflammation and hepatic insulin resistance [42]. Moreover, severe hepatic dysfunction in cirrhotic patients may mask typical metabolic responses, further complicating this relationship [33].

Multivariable modeling consistently identified BMI (≥30 kg/m^2^) as the strongest modifiable risk factor for T2DM, underscoring the central role of obesity and metabolic syndrome in diabetes pathogenesis. Unlike some prior work, age was not an independent predictor of T2DM after adjustment for BMI, further underscoring the dominant role of adiposity on post-HCV metabolic risk [43,44]. Additionally, HCV genotype distribution varies geographically and may influence diabetes risk. A Southeast Asian study reported genotype 3 as an independent predictor of T2DM in HCV-infected patients [4], whereas genotype 1b predominated in our Taiwanese cohort [31]. Therefore, regional genotype differences may partly contribute to heterogeneous metabolic outcomes and should be considered when interpreting the association between antiviral therapy and incident T2DM.

Notably, genotype-related metabolic effects may partly explain the discrepancy between crude incidence differences and the absence of statistically significant associations after adjustment. Although crude T2DM incidence appeared lower in the DAA-treated cohort, the difference was attenuated after adjustment for baseline metabolic and clinical variables. This finding suggests that patient-related metabolic factors, particularly BMI, may have contributed more substantially to post-treatment diabetes risk than antiviral regimen alone.

The observed differences between IFN- and DAA-treated cohorts should be interpreted with caution because the two treatment modalities were administered during different clinical eras. Although IPTW and sensitivity analyses were performed to reduce baseline imbalance, residual confounding related to treatment period, healthcare accessibility, and evolving clinical practice patterns cannot be completely excluded.

In the present study, no significant difference in incident T2DM was observed between IFN- and DAA-treated patients after stratification by SVR status. Therefore, the apparent divergence in crude T2DM trajectories should not be interpreted as evidence that the method of achieving HCV cure directly modifies diabetes risk. One plausible explanation is that SVR removes the HCV-related component of metabolic stress, such as chronic inflammation and IR, after which future T2DM risk is mainly determined by pre-existing host factors. This interpretation is consistent with our finding that BMI ≥ 30 kg/m^2^, rather than antiviral modality or SVR status, remained independently associated with incident T2DM. Differences in cumulative incidence may therefore reflect treatment-era differences, follow-up duration, and residual confounding rather than a true biological difference between IFN- and DAA-mediated cures.

Although the present study did not evaluate cardiovascular outcomes, the broader translational context of HCV eradication is worth noting. Previous studies have suggested that successful viral clearance—particularly through DAA therapy—may confer extrahepatic benefits beyond glucose metabolism, including potential reductions in acute coronary syndrome, ischaemic stroke, and carotid atherosclerosis progression in high-risk populations [1]. The mechanisms proposed include resolution of chronic HCV-mediated inflammation, improvements in endothelial function, and attenuation of insulin resistance. While the cardiovascular implications of DAA therapy were beyond the scope of this study, these observations support the notion that HCV eradication may have broad systemic metabolic effects [28]. Future prospective studies incorporating both metabolic and cardiovascular endpoints, with longer follow-up periods and standardized adjustment for treatment-era differences, will be needed to establish the full extrahepatic benefit profile of contemporary antiviral regimens.

This study has several limitations. Its retrospective design and reliance on administrative and electronic health record data may introduce selection and information biases, and residual confounding cannot be fully excluded despite adjustment for key covariates. The shorter follow-up duration in the DAA group may underestimate long-term outcomes relative to IFN-treated patients. Unavailable data on lifestyle factors and laboratory indices of glucose metabolism, such as HOMA-IR and fasting glucose, limit mechanistic interpretation. Additionally, the study population was predominantly Taiwanese, potentially limiting generalizability to other populations. Some subgroup analyses were limited by small event numbers, resulting in wide confidence intervals and reduced statistical precision.

## 5. Conclusions

Among patients with chronic hepatitis C who achieved sustained virologic response following antiviral therapy, crude incidence rates of type 2 diabetes mellitus differed between IFN- and DAA-treated cohorts. However, these differences were not consistently significant after adjustment for baseline metabolic and clinical factors. While viral eradication may be associated with favorable metabolic trends, the present findings do not establish a causal protective effect against incident T2DM. Increased BMI remained an independent predictor of post-treatment diabetes risk, underscoring the importance of long-term metabolic monitoring after HCV eradication. Further prospective studies with longer follow-up and improved control of era-related confounding are warranted to clarify the metabolic impact of contemporary HCV therapies.

## Figures and Tables

**Table 1:** Baseline demographic and clinical characteristics of study participants.

	Overall (*N* = 2489)		DAA-Based (*N* = 1708)		IFN-Based (*N* = 781)		*p*-Value
Age, mean (SD)	59.31	(12.97)	60.20	(13.01)	57.35	(12.68)	<0.001 *
BMI, kg/m^2^ mean (SD)	24.48	(3.97)	24.25	(3.94)	24.96	(4.01)	0.02 *
Gender, *N* (%)							<0.001 *
Male	1092	(43.87)	699	(40.93)	393	(50.32)	
Female	1397	(56.13)	1009	(59.07)	388	(49.68)	
HCV genotype, *N* (%)							
1a	24	(0.97)	21	(1.23)	3	(0.38)	0.05 *
1b	612	(24.61)	575	(33.68)	37	(4.74)	<0.001 *
1 + 2	10	(0.4)	6	(0.35)	4	(0.51)	0.52
HCV RNA, log10IU/mL mean (SD)	6.53	(6.53)	6.53	(6.66)	6.54	(6.82)	0.82
ALT, IU/L (SD)	83.77	(101)	67.07	(75.73)	116.50	(131.8)	<0.001 *
AST, IU/L (SD)	66.42	(70.1)	57.76	(60.26)	83.63	(83.81)	<0.001 *
Total bilirubin, mg/dL (SD)	0.72	(0.43)	0.69	(0.4)	0.79	(0.49)	<0.001 *
INR	1.05	(0.11)	1.05	(0.12)	1.07	(0.08)	<0.001 *
Therapy duration at weeks, *N* (%)							
≤8	1290	(51.83)	1147	(67.15)	143	(18.31)	<0.001 *
12	701	(28.16)	539	(31.56)	162	(20.74)	<0.001 *
24	306	(12.29)	22	(1.29)	284	(36.36)	<0.001 *
36	20	(0.8)	0	(0)	20	(2.56)	<0.001 *
48	153	(6.15)	0	(0)	153	(19.59)	<0.001 *
>48	19	(0.76)	0	(0)	19	(2.43)	<0.001 *
RBV combined	779	(31.3)	0	(0)	779	(99.74)	<0.001 *
Complications, *N* (%)							
Cirrhosis	75	(3.01)	49	(2.87)	26	(3.33)	0.53
Hepatic fibrosis	306	(12.29)	208	(12.18)	98	(12.55)	0.79
Peritoneal Dialysis	15	(0.6)	13	(0.76)	2	(0.26)	0.17
CKD	149	(5.99)	126	(7.38)	23	(2.94)	<0.001 *
Liver tumor	201	(8.08)	132	(7.73)	69	(8.83)	0.34
Digestive system neoplasms	367	(14.74)	253	(14.81)	114	(14.6)	0.90
Hyperlipidaemia	359	(14.42)	200	(11.71)	159	(20.36)	<0.001 *
Hypertension	619	(24.87)	450	(26.35)	169	(21.64)	0.01 *
Peptic ulcer	1060	(42.59)	612	(35.83)	448	(57.36)	<0.001 *
Gastric ulcer	741	(29.77)	492	(28.81)	249	(31.88)	0.12
Constipation	521	(20.93)	317	(18.56)	204	(26.12)	<0.001 *
Dizziness and giddiness	441	(17.72)	290	(16.98)	151	(19.33)	0.16
Functional dyspepsia	399	(16.03)	239	(13.99)	160	(20.49)	<0.001 *
GERD with esophagitis	425	(17.08)	269	(15.75)	156	(19.97)	0.01 *
Acute abdomen	424	(17.03)	265	(15.52)	159	(20.36)	<0.001 *
UTI	331	(13.3)	236	(13.82)	95	(12.16)	0.28
Sleep disorder	417	(16.75)	199	(11.65)	218	(27.91)	<0.001 *
Anxiety disorder	351	(14.1)	178	(10.42)	173	(22.15)	<0.001 *
Peptic ulcer with hemorrhage	342	(13.74)	155	(9.07)	187	(23.94)	<0.001 *
Irritable bowel syndrome	413	(16.59)	263	(15.4)	150	(19.21)	0.02 *
Flatulence	239	(9.6)	100	(5.85)	139	(17.8)	<0.001 *
Medication 12 months before starting treatment, *N* (%)							
Silymarin	1163	(46.73)	738	(43.21)	425	(54.42)	<0.001 *
Acetaminophen 500 mg	979	(39.33)	419	(24.53)	560	(71.7)	<0.001 *
Famotidine 20 mg	784	(31.5)	523	(30.62)	261	(33.42)	0.16
Fursultiamine 50 mg, Riboflavin 5 mg	565	(22.7)	369	(21.6)	196	(25.1)	0.06
Proheparum tab	440	(17.68)	153	(8.96)	287	(36.75)	<0.001 *
Pantoprazole 40 mg	409	(16.43)	277	(16.22)	132	(16.9)	0.68
Dimethylpolysiloxane 40 mg	409	(16.43)	287	(16.8)	122	(15.62)	0.48
Mosapride citrate 5 mg	372	(14.95)	246	(14.4)	126	(16.13)	0.28
Sennosides 20 mg	300	(12.05)	226	(13.23)	74	(9.48)	0.01 *

SD, standard deviation; BMI, Body Mass Index; IFN, interferon; DAA, direct-acting antiviral agent; RBV, ribavirin; CKD, Chronic kidney disease; GERD, Gastro-esophageal reflux disease; UTI, Urinary tract infection. * Significant difference (*p* < 0.05).

**Table 2:** Incidence of type 2 diabetes mellitus by antiviral regimen and liver function.

	Event	*N*	Average (Years)	SD	Cumulative Incidence (%)
All patients					
Overall	96	2489	2.56	1.86	3.86
IFN-based	54	781	4.53	1.89	6.91
DAA-based	42	1708	1.66	0.91	2.46
Normal liver function					
Overall	72	2074	2.55	1.92	3.36
IFN-based	42	631	4.65	1.90	6.24
DAA-based	30	1443	1.59	0.87	2.04
Fibrosis					
Overall	23	306	1.78	1.06	7.52
IFN-based	11	98	2.26	1.06	11.22
DAA-based	12	208	1.35	0.89	5.77
Cirrhosis					
Overall	1	49	1.12	-	2.04
IFN-based	1	13	1.12	-	7.14
DAA-based	0	36	0	-	0

IFN, interferon; DAA, direct-acting antiviral agent; SD, standard deviation.

**Table 3:** Subgroup analysis of type 2 diabetes mellitus incidence according to different DAA regimens.

Antiviral Regimens	HCV Genotype	Total		1 Year		2 Years		3 Years		*p*-Value (Trend Test)
		*N*	(%)	*N*	(%)	*N*	(%)	*N*	(%)	
Daclatasvir/Asunaprevir	1a, 1b	4/24	(16.67)	2	(8.33)	3	(12.50)	4	(16.67)	<0.001 *
Dasabuvir/Ombitasvir/Paritaprevir/Ritonavir	1a, 1b, 4	7/164	(4.27)	1	(0.61)	3	(1.83)	7	(4.27)	<0.001 *
Ledipasvir/Sofosbuvir	1, 2, 4, 5, 6	14/363	(3.86)	7	(1.93)	14	(3.86)	14	(3.86)	<0.001 *
Elbasvir/Grazoprevir	1a, 1b, 4	14/425	(3.29)	5	(1.18)	11	(2.59)	14	(3.29)	<0.001 *
Glecaprevir/Pibrentasvir	pan-GT	2/412	(0.49)	1	(0.24)	2	(0.49)	2	(1.00)	0.08
Sofosbuvir/Velpatasvir	pan-GT	1/318	(0.31)	1	(0.31)	1	(0.31)	1	(0.31)	0.17
*p*-value (Chi-square)		<0.001 *		1.00		0.09		0.004 *		

* Significant difference (*p* < 0.05).

## Data Availability

Data available within the article or its Supplementary Materials.

## Supplementary Materials

The following supporting information can be downloaded at: https://www.mdpi.com/article/10.3390/biomedicines14061352/s1. Figure S1: Study procedures for evaluating the risk of type 2 diabetes mellitus; Figure S2: Virological response rates; Figure S3: Comparison of type 2 diabetes mellitus incidence between interferon-based and direct-acting antiviral–based treatment cohorts: (A) overlap of type 2 diabetes incidence over time; (B) Kaplan–Meier curve for type 2 diabetes incidence among patients achieving sustained virologic response; Figure S4: Estimated hemoglobin A1c change before and after treatment; Table S1: Diagnostic criteria for comorbidities; Table S2: Standardized mean differences before and after propensity score weighting for type 2 diabetes risk; Table S3: Incidence rate ratio analysis of type 2 diabetes and virological outcomes stratified by liver function status; Table S4: Hazard ratios for type 2 diabetes mellitus by liver function and virologic response; Table S5: Cumulative incidence of type 2 diabetes mellitus over time (1–5 years) by treatment; Table S6: Hazard ratios for type 2 diabetes mellitus stratified by age and body mass index; Table S7: Sensitivity analysis of type 2 diabetes mellitus incidence in the propensity score–matched cohort.

## Abbreviations

The following abbreviations are used in this manuscript:

BMI	Body Mass Index
CI	Confidence Interval
CKD	Chronic Kidney Disease
DAA	Direct-Acting Antiviral
DRD	Ditmanson Research Database
GT	Genotype
HbA1c	Glycated Hemoglobin
HCV	Hepatitis C Virus
HR	Hazard Ratio
HTN	Hypertension
ICD	International Classification of Diseases
IFN	Interferon
IR	Insulin Resistance
IRR	Incidence Rate Ratio
IPTW	Inverse Probability of Treatment Weighting
PD	Parkinson’s Disease
PS	Propensity Score
RBV	Ribavirin
SMD	Standardized Mean Difference
SVR	Sustained Virologic Response
T2DM	Type 2 Diabetes Mellitus
TNF-α	Tumor Necrosis Factor Alpha
