# Supplementary material for: Long-Term Risk Trajectories of Diabetes Differ After Direct-Acting Antiviral and Interferon Therapy in Chronic Hepatitis C: A Real-World Cohort Study
Source: Biomedicines. 2026 Jun 15;14(6):1352. doi: 10.3390/biomedicines14061352 (PMC13297605; doi:10.3390/biomedicines14061352)
Supplement: Supplementary file 1 [file biomedicines-14-01352-s001.zip › biomedicines-4287128-supplementary.pdf]

Long-Term Risk Trajectories of Diabetes Differ After Direct-Acting Antiviral and Interferon Therapy in Chronic Hepatitis C: A Real-World Cohort Study

Hsuan-Yu Hung [1,2], Wei-Liang Hung [3] and Chung-Yu Chen [1,4,*]

[1] School of Pharmacy, College of Pharmacy, Kaohsiung Medical University, Kaohsiung 80708, Taiwan; ameeyo36@gmail.com
[2] Department of Pharmacy, Ditmanson Medical Foundation Chia-Yi Christian Hospital, Chiayi 60002, Taiwan
[3] Department of Medicine, Division of Nephrology, Zuoying Armed Forces General Hospital, Kaohsiung 81342, Taiwan; lydership@gmail.com
[4] Master Program in Clinical Pharmacy, School of Pharmacy, Kaohsiung Medical University, Kaohsiung 80756, Taiwan
* Correspondence: jk2975525@hotmail.com; Tel.: +886-7-3121101 (ext. 2375)

Contents

Table S1. Diagnostic criteria for comorbidities 2

Table S2. Standardized Mean Differences Before and After Propensity Score Weighting for Type 2 diabetes Risk 5

Table S3. Incidence rate ratio analysis of type 2 diabetes and virological outcomes stratified by liver function status 7

Table S4. Hazard ratios for type 2 diabetes mellitus by liver function and virologic response 8

Table S5. Cumulative incidence of type 2 diabetes mellitus over time (1–5 years) by treatment 9

Table S6. Hazard ratios for type 2 diabetes mellitus, stratified by age and body mass index 10

Table S7. Sensitivity analysis of type 2 diabetes mellitus incidence in the propensity score–matched cohort 11

Figure S1. Study procedures for evaluating the risk of type 2 diabetes mellitus 12

Figure S2. Virological response rates 12

Figure S3. Comparison of type 2 diabetes mellitus incidence between interferon-based and direct-acting antiviral–based treatment cohorts: (A). Overlap of type 2 diabetes incidence time; (B). Kaplan-Meier curve for type 2 diabetes incidence with achieved sustained virologic response 13

Figure S4. Estimated hemoglobin A1c change before and after treatment 14

Table S1. Determine the diagnosis for comorbidities.

Category	Diagnosis	ICD-9-CM	ICD-10-CM
	Chronic hepatitis C	070.44, 070.54, 070.70, 070.71, 070.41, 070.51, V02.62	B17.10; B17.11; B18.2; Z22.52; B19.20; B19.21; Z22.52
	Type 2 diabetes mellitus	250.00, 250.02, 250.10, 250.12, 250.20, 250.22, 250.30, 250.32, 250.40, 250.42, 250.50, 250.52, 250.60, 250.62, 250.70, 250.72, 250.80, 250.82, 250.90, 250.92, 357.2, 362.01, 362.02, 366.41, 583.81	E11.00, E11.01, E11.21, E11.22, E11.29, E11.311, E11.319, E11.321, E11.329, E11.331, E11.339, E11.341, E11.349, E11.351, E11.359, E11.36, E11.39, E11.40, E11.41, E11.42, E11.43, E11.44, E11.49, E11.51, E11.52, E11.59, E11.610, E11.618, E11.620, E11.621, E11.622, E11.628, E11.630, E11.638, E11.641, E11.649, E11.65, E11.69, E11.8, E11.9
	Liver tumor	155, 155.1	C22.0, C22.2, C22.3, C22.4, C22.7, C22.8, C22.1
	Hepatic fibrosis	571.5	K74.00, K74.01, K74.02
	Cirrhosis	571.5, 571.6, 571.8, 571.9, 571.2	K74.0, K74.1, K74.2, K74.60, K74.69, K74.3, K74.4, K74.5, K76.0, K70.30, K70.31, K70.32, K70.38, K70.39
	Hepatocellular Carcinoma	155.0	C22.0
	End Stage Liver Disease	571.2, 571.5, 571.6	K70.2-K70.31, K74.1-K74.69
Exclusion Criteria	Type 1 diabetes mellitus	250.01, 251.03, 250.11, 250.13, 250.21, 250.23, 250.31, 250.33, 250.41, 250.43, 250.51, 250.53, 250.61, 250.63, 250.71, 250.73, 250.81, 250.83, 250.91, 250.93, 357.2, 366.41, 583.81	E10.10 E10.11 E10.21 E10.22 E10.29 E10.311, E1.319, E10.321, E10.329, E10.331 E10.339, E10.341, E10.349, E10.351, E10.359 E10.36, E10.39, E10.40, E10.41, E10.42, E10.43, E10.44, E10.49, E10.51, E10.52, E10.59, E10.610, E10.618, E10.620, E10.621, E10.622, E10.628, E10.630, E10.638, E10.641 E10.649, E10.65, E10.69, E10.8, E10.9
	Hepatitis B virus	70.3, 70.3, 70.31, 70.32, 70.33, V02.61, 571.49	B16.1, B16.2, B18.0, B18.1
	Human immunodeficiency	42	B20.X, B21.X, B22.X, B23.X, B24.X, Z21

Category	Diagnosis	ICD-9-CM	ICD-10-CM
	liver transplant	V42.7	Z94.4
	kidney transplant	V42.0	Z940
Comorbidities	Coronary Artery Disease	4140.1	I70, I21, I22, I24.0, I24.8, I24.9, I25.0, I25.1, I25.2
	Obesity	2780	E66
	Smoking	305.1, V15.82	F17.2, Z72.0
	Hypothyroidism	244.9	E03, E02, E89.0
	Fatty liver	571.8, 571.8	K76.0, K70.0 K75.8 K74.6
	Insulin resistance	277.7	E88.818
	Atherosclerosis	440.9	I70.90
	Myocardial dysfunction	428.0	I50.9
	Cognitive impairment	780.93	R41.9
	Fibromyalgia syndrome	729.1	M79.7
	Chronic kidney disease	40311, 40391, 40412, 40492, 585, 586, V420, V451, V560, V568	N18.1, N18.2, N18.3, N18.4, N18.5, N18.6, N18.9, N19
	diabetic nephropathy	583.81, 250.4, 250.41, 250.42, 250.43, 251.8	E08.21, E09.21, E10.21, E11.21, E13.21
	Chronic Obstructive Pulmonary Disease	491.22, 491.21, 491.20	J44
	Hyperlipidaemia	272, 272.1, 272.2, 272.3, 272.4, 272.5, 272.8, 272.9, 759.89	E78.0, E78.1, E78.2, E78.3, E78.4, E78.5, E78.6, E78.79, E78.89, E78.70, E78.9, E78.71, E78.72
	Hypertension	401, 401.1, 401.9, 402, 402.1, 402.9, 402.01, 402.11, 402.91, 403.01, 403.11, 403.91, 403, 403.1, 403.9, 404.01, 404.11, 404.91, 404, 404.1, 404.9, 404.02, 404.12, 404.92, 404.03, 404.13, 404.93, 405.01, 405.11, 405.91, 405.99, 405.09, 405.19	I10, I11.9, I11.0, I12.0, I12.9, I13.0, I13.10, I13.11, I13.2, I15.0, I15.1, I15.2, I15.8, I15.9, I16.0, I16.1, I16.9
	Essential mixed cryoglobulinemia	273.2	D89.1
	Monoclonal gammopathy	273.1	D47.2
	B-cell non-Hodgkin lymphoma	202.8x	C83.0 C85.1 C83.3

Category	Diagnosis	ICD-9-CM	ICD-10-CM
	Leukocytoclastic vasculitis	695.2	L95.8
	Porphyria cutanea tarda	277.1	E80.1
	Lichen planus	697.0	L43.8
	Membranoproliferative glomerulonephritis	583.2	N05.3 N05.5
	IgA nephropathy	583.1	N02.8
	Autoimmune hemolytic anemia	283.0	D59.1
	Immune thrombocytopenic purpura	287.31	D69.3
	Autoimmune thyroiditis	245.2	E06.3
	Sicca syndrome	710.2	M35.0
	coronary artery disease	410–414	I25.1 A279
	Arrhythmia	427.0, 427.1, 427.2, 427.3, 427.4, 427.5, 427.9, 427.89	I49.9, I47.0, I47.1, I47.2, I47.9, I48.0, I48.1, I48.2, I48.3, I48.4, I48.91, I48.92, I49.01, I49.02
	Heart Failure	428, 428.0, 428.1, 428.2, 428.3, 428.4, 428.9	I50, I50.9, I50.1, I50.20, I50.21, I50.22, I50.23, I50.30, I50.31, I50.32, I50.33, I50.40, I50.41, I50.42, I50.43, I50.8
	cerebrovascular accidents	430–438	I60-I69
	Digestive system neoplasms	239, 235.2, 235.3, 25.5	D49.0, D37.1, D37.2, D37.3, D37.4, D37.5, D37.6, D37.8, D37.9

Table S2. Standardized Mean Differences Before and After Propensity Score Weighting for Type 2 diabetes Risk.

Standardized Mean Difference (Treated − Control)						
**Variable**	**Stratum**	**Observations**	**Mean Difference**	**Standard Deviation**	**Standardized Difference**	**% Reduction**
**Logit of Propensity Score**	Overall	0.12754	0.35917	0.35509		0.9195
	Region	0.12754		0.35509	0	0.9195
	Weighted	-0.00179		-0.00498	98.6	0.8923
**Age**	Overall	0.66587	12.13382	0.05488		1.4101
	Region	0.66587		0.05488	0	1.4101
	Weighted	-0.0438		-0.00361	93.42	1.408
**BMI**	Overall	-0.7184	4.02499	-0.17848		0.9153
	Region	-0.7184		-0.17848	0	0.9153
	Weighted	0.01541		0.00383	97.86	0.9543
**Gender**	Overall	0.06176	0.49869	0.12385		0.9907
	Region	0.06176		0.12385	0	0.9907
	Weighted	-0.00159		-0.0032	97.42	1.0004
**Digestive system neoplasms**	Overall	-0.0509	0.39968	-0.12735		1.2109
	Region	-0.0509		-0.12735	0	1.2109
	Weighted	0.00273		0.00682	94.64	0.9904
**Liver tumor**	Overall	0.04309	0.38632	0.11153		0.8325
	Region	0.04309		0.11153	0	0.8325
	Weighted	0.00058		0.00151	98.64	0.9975
**Peritoneal Dialysis**	Overall	0.00171	0.06717	0.02552		0.6832
	Region	0.00171		0.02552	0	0.6832
	Weighted	-0.00001		-0.00022	99.13	1.0036
**Hyperlipidaemia**	Overall	0.04701	0.37539	0.12523		0.802
	Region	0.04701		0.12523	0	0.802
	Weighted	-0.00092		-0.00245	98.04	1.0046
**Chronic Kidney Disease**	Overall	-0.02097	0.27608	-0.07597		1.2591
	Region	-0.02097		-0.07597	0	1.2591

Standardized Mean Difference (Treated − Control)						
**Variable**	**Stratum**	**Observations**	**Mean Difference**	**Standard Deviation**	**Standardized Difference**	**% Reduction**
	Weighted	0.00064		0.00233	96.94	0.9932
**Hepatic fibrosis**	Overall	0.03572	0.41879	0.0853		0.8948
	Region	0.03572		0.0853	0	0.8948
	Weighted	0.00172		0.0041	95.19	0.9946
**Hypertension**	Overall	-0.04531	0.48824	-0.09281		1.0409
	Region	-0.04531		-0.09281	0	1.0409
	Weighted	-0.00196		-0.00402	95.67	1.0017
**Cirrhosis**	Overall	0.00024	0.16929	0.0014		0.9922
	Region	0.00024		0.0014	0	0.9922
	Weighted	-0.00093		-0.0055	0	1.0322

Table S3. Incidence rate ratio analysis of type 2 diabetes and virological outcomes stratified by liver function status.

**Characteristics**	**Overall**		**DAA-based**		**IFN-based**		**IRR (95% CI)**	**p-Value**
	**N**	**(%)**	**N**	**(%)**	**N**	**(%)**		
All patients (N=2489)	96	(3.86)	42	(2.46)	54	(6.91)	0.97 (0.63-1.48)	0.97
SVR	41	(2.39)	28	(1.87)	13	(5.88)	1.13 (0.57-2.37)	0.86
Virologic Failure	3	(4.55)	1	(5.26)	2	(4.26)	1.91 (0.03-36.63)	1.00
Relapse	1	(3.45)	0	(0)	1	(5.88)	-	-
Normal liver function (N=2074)	72	(3.36)	30	(2.04)	42	(6.24)	0.96(0.60-1.52)	0.84
SVR	35	(2.3)	24	(1.81)	11	(5.56)	1.20 (0.58-2.44)	0.62
Virologic Failure	3	(5.88)	1	(8.33)	2	(5.13)	0.64 (0.01-12.64)	1.00
Relapse	1	(4.00)	0	(0)	1	(6.67)	-	
Fibrosis (N=306)	23	(7.52)	12	(5.77)	11	(11.22)	2.22 (0.73-7.1)	0.19
SVR	5	(3.03)	4	(2.76)	1	(5.00)	2.71 (0.23-143.39)	0.70
Virologic Failure	-	-	-	-	-	-	-	-
Relapse	-	-	-	-	-	-	-	-
Cirrhosis (N=49)	1	(2.04)	0	(0)	1/14	(7.14)	-	-
SVR	1	(2.94)	0	(0)	1/4	(25.00)	-	-
Virologic Failure	-	-	-	-	-	-	-	-
Relapse	-	-	-	-	-	-	-	-

IFN, interferon; DAA, direct-acting antiviral agent; SVR, sustained virologic response after treatment; IRR, incidence rate ratio; 95% CI, confidence interval. * Significant difference ($P < 0.05$). Patients with fibrosis may also have been classified as having cirrhosis; therefore, subgroup totals may not exactly equal the overall number of patients.

Table S4. Hazard ratios for type 2 diabetes mellitus by liver function and virologic response.

Characteristics	Univariable HR (95% CI)	P-value	Multivariable adjusted HRa (95% CI)	P-value
All patients (N=2489)	0.97 (0.62 - 1.52)	0.88	0.84 (0.51 - 1.38)	0.49
SVR	1.14 (0.51 - 2.54)	0.75	1.29 (0.56 - 2.98)	0.55
Virologic Failure	1.21 (0.11 - 13.35)	0.88	1.02 (0.08 - 13.76)	0.99
Relapse	-	-	-	-
Normal liver function (N=2074)	0.97 (0.57 - 1.65)	0.90	0.95 (0.54 - 1.69)	0.87
SVR	1.2 (0.5 - 2.87)	0.68	1.42 (0.57 - 3.52)	0.45
Virologic Failure	1.58 (0.14 - 17.46)	0.71	-	-
Relapse	-	-	-	-
Fibrosis (N=306)	0.88 (0.37 - 2.09)	0.77	0.48 (0.17 - 1.41)	0.18
SVR	4.96 (0.2 - 124.58)	0.33	-	-
Virologic Failure	-	-	-	-
Relapse	-	-	-	-
Cirrhosis (N=49)	-	-	-	-
SVR	-	-	-	-
Virologic Failure	-	-	-	-
Relapse	-	-	-	-

IFN, interferon; DAA, direct-acting antiviral agent; SVR, sustained virologic response after treatment; IRR, incidence rate ratio; 95% CI, confidence interval.

* Significant difference (P < 0.05).

[a] multivariable adjusted hazard ratios (HR) accounting for HCV genotype1b.

Subgroup categories were not mutually exclusive. Patients with fibrosis may also have been classified as having cirrhosis; therefore, subgroup totals may not exactly equal the overall number of patients.

Table S5. Cumulative incidence of type 2 diabetes mellitus over time (1–5 years) by treatment.

Treatment Group	1 year	2 years	3 years	4 years	5 years	p-value (Trend Test)
	N (%)	N (%)	N (%)	N (%)	N (%)	
Overall	25 (1.00%)	59 (2.37%)	79 (3.17%)	86 (3.46%)	90 (3.62%)	<0.001*
IFN-based	8 (1.02%)	25 (3.20%)	37 (4.74%)	44 (5.63%)	48 (6.15%)	<0.001*
DAA-based	17 (1.00%)	34 (1.99%)	42 (2.46%)	42 (2.46%)	42 (2.46%)	0.001*
p-value (Chi-square)	1.00	0.087	0.004*	<0.001*	<0.001*	

* Significant difference (P < 0.05).

Table S6. Hazard ratios for type 2 diabetes mellitus, stratified by age and body mass index

	Overall		DAA-based		IFN-based		Univariable HR (95% CI)	p-value	Multivariable adjusted HR[a] (95% CI)	p-value
	N	(%)	N	(%)	N	(%)				
Age group (years)							0.89 (0.57 - 1.4)	0.62	0.88 (0.47 - 1.66)	0.69
<20	4	(0.16)	1	(0.06)	3	(0.38)	-	-	-	
≥20 to <40	186	(7.47)	114	(6.67)	72	(9.22)	0.7 (0.24 - 2.07)	0.62	0.71 (0.24 - 2.11)	0.54
≥40 to <55	650	(26.11)	429	(25.12)	221	(28.3)	Reference		Reference	
≥55 to <65	762	(30.61)	511	(29.92)	251	(32.14)	1.7 (0.97 - 2.99)	0.52	1.7 (0.97 - 2.98)	0.07
≥65 to <75	574	(23.06)	397	(23.24)	177	(22.66)	1.59 (0.86 - 2.94)	0.14	1.6 (0.87 - 2.95)	0.13
≥75	313	(12.58)	256	(14.99)	57	(7.3)	1.97 (0.96 - 4.07)	0.07	1.89 (0.91 - 3.92)	0.09
BMI, kg/m^2							0.92 (0.49 - 1.72)	0.80	0.84 (0.45 - 1.58)	0.59
<18.5	44	(5.52)	31	(5.7)	13	(5.14)	0.47 (0.06 - 3.47)	0.46	0.48 (0.07 - 3.59)	0.48
≥18.5 to <25	432	(54.2)	304	(55.88)	128	(50.59)	Reference		Reference	
≥25 to <30	255	(31.99)	169	(31.07)	86	(33.99)	1.32 (0.69 - 2.53)	0.40	1.35 (0.71 - 2.58)	0.36
≥30	66	(8.28)	40	(7.35)	26	(10.28)	2.47 (1.08 - 5.61)	0.03*	2.63 (1.15 - 6.01)	0.02*

IFN, interferon; DAA, direct-acting antiviral agent; 95% CI, confidence interval.

[a] multivariable adjusted hazard ratios (HR) accounting for HCV genotype1b.

* Significant difference (P < 0.05).

Table S7. Sensitivity analysis of type 2 diabetes mellitus incidence in the propensity score–matched cohort.

	Event	Total	Cumulative Incidence (%)	HR (95% CI)
Overall	50	864	5.79	
IFN-based	23	359	6.41	0.81 (0.43-1.53)
DAA-based	27	505	5.35	

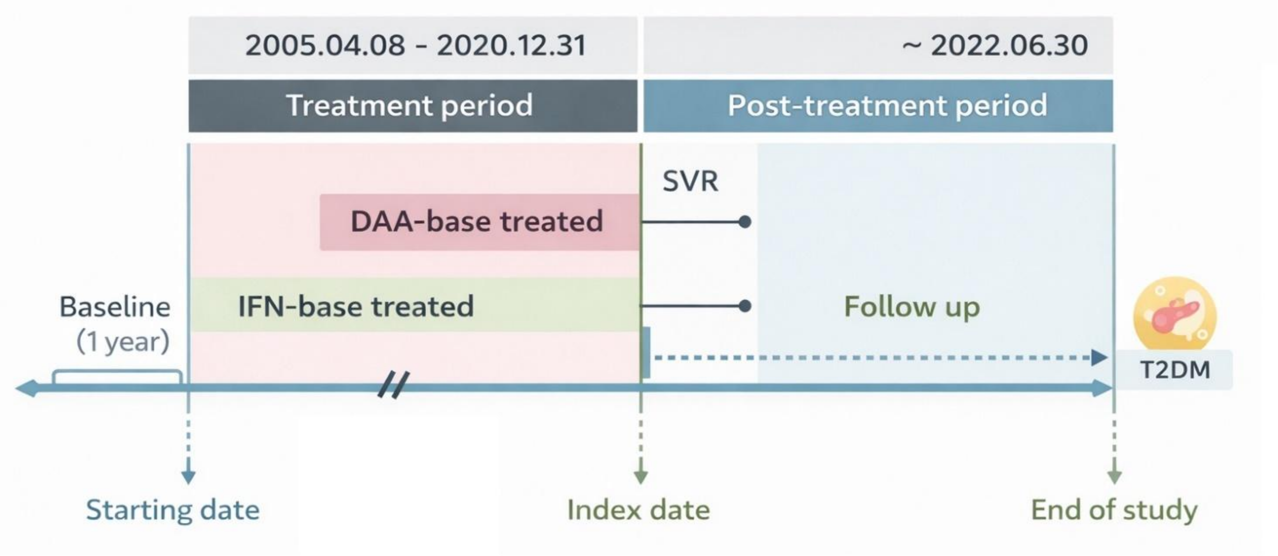

Figure S1. Study procedures for evaluating the risk of type 2 diabetes mellitus.

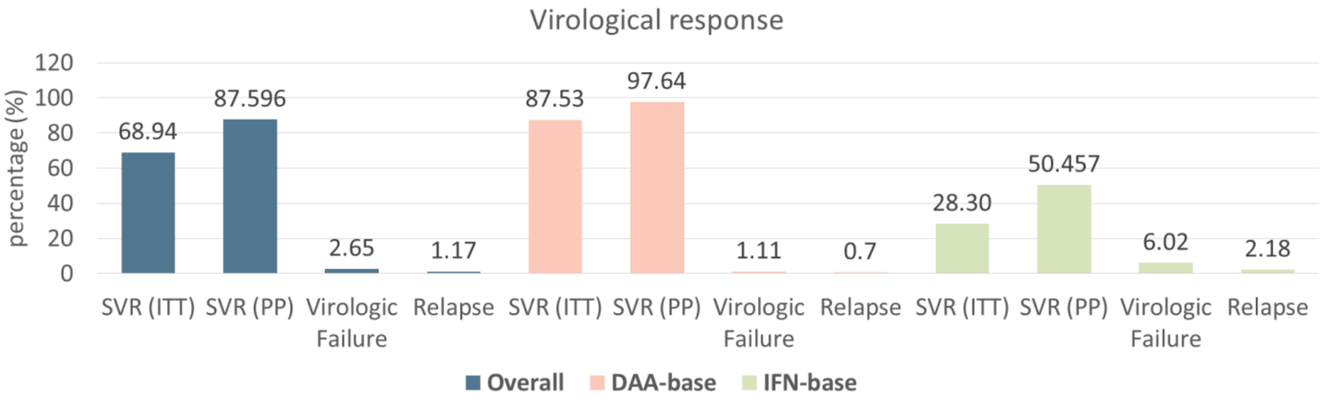

Figure S2. Virological response rates.

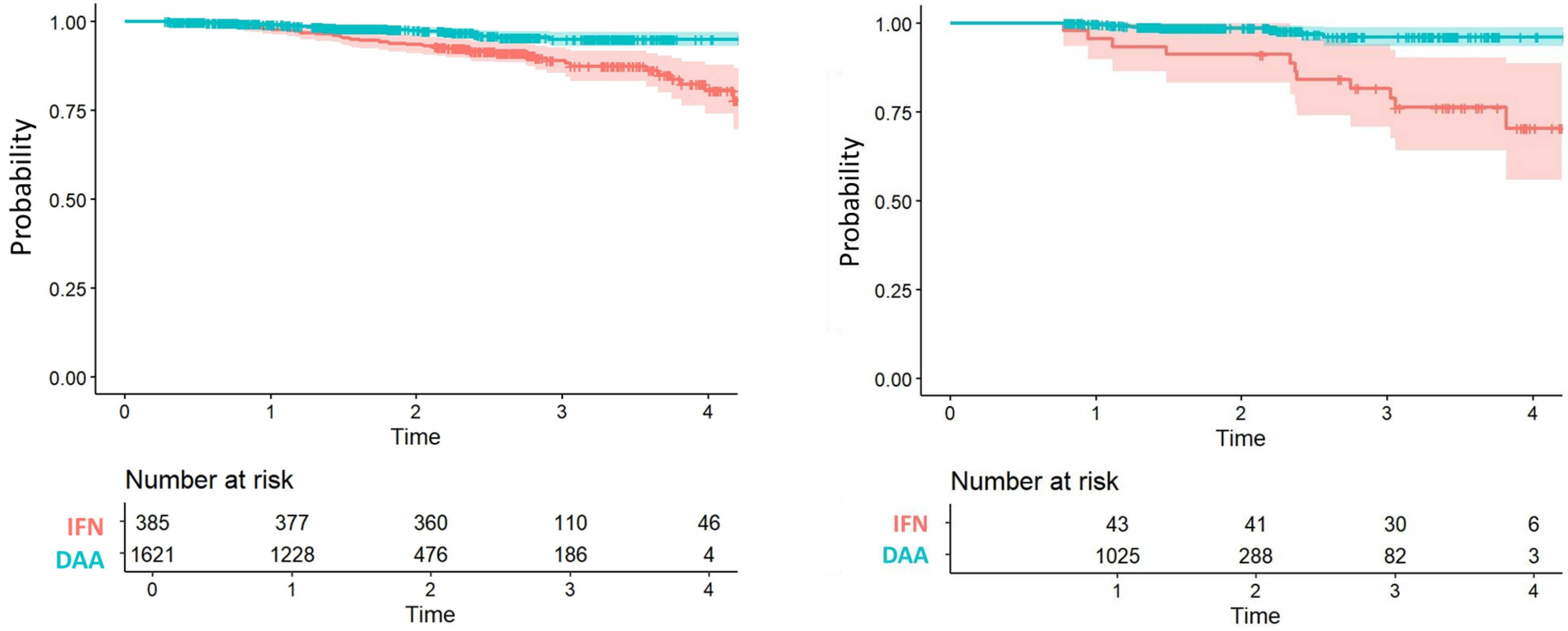

(A) **(B)**

Figure S3. Comparison of type 2 diabetes mellitus incidence between interferon-based and direct-acting antiviral–based treatment cohorts: (**A**). Overlap of type 2 diabetes incidence time; (**B**). Kaplan-Meier curve for type 2 diabetes incidence with achieved sustained virologic response

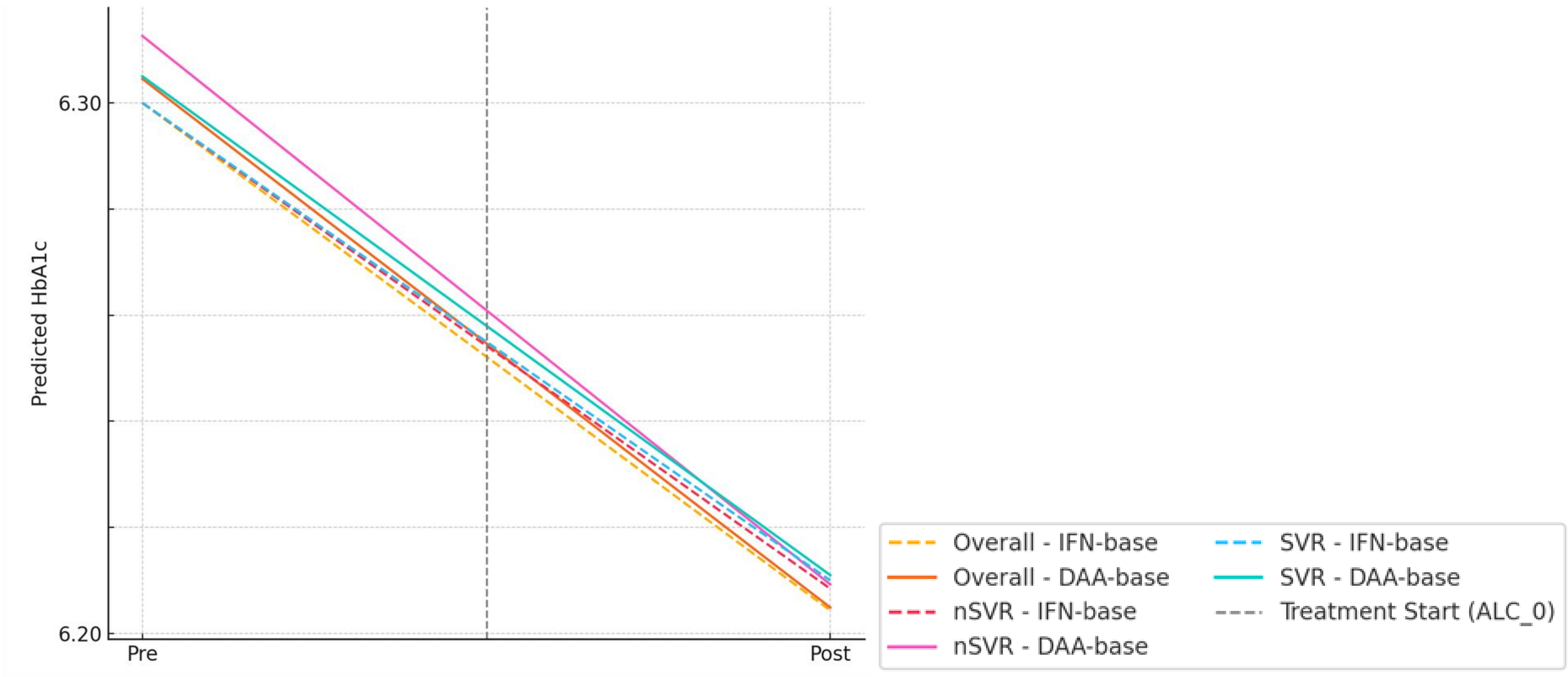

Figure S4. Estimated hemoglobin A1c change before and after treatment.

.
